# Detection of Transgenerational Spermatogenic Inheritance of Adult Male Acquired CNS Gene Expression Characteristics Using a *Drosophila* Systems Model

**DOI:** 10.1371/journal.pone.0005763

**Published:** 2009-06-02

**Authors:** Abhay Sharma, Priyanka Singh

**Affiliations:** Institute of Genomics and Integrative Biology, Council of Scientific and Industrial Research, Delhi, India; Brunel University, United Kingdom

## Abstract

Available instances of inheritance of epigenetic transgenerational phenotype are limited to environmental exposures during embryonic and adult gonadal development. Adult exposures can also affect gametogenesis and thereby potentially result in reprogramming of the germline. Although examples of epigenetic effects on gametogenesis exist, it is notable that transgenerational inheritance of environment-induced adult phenotype has not yet been reported. Epigenetic codes are considered to be critical in neural plasticity. A *Drosophila* systems model of pentylenetetrazole (PTZ) induced long-term brain plasticity has recently been described. In this model, chronic PTZ treatment of adult males causes alterations in CNS transcriptome. Here, we describe our search for transgenerational spermatogenic inheritance of PTZ induced gene expression phenotype acquired by adult *Drosophila* males. We generated CNS transcriptomic profiles of F_1_ adults after treating F_0_ adult males with PTZ and of F_2_ adults resulting from a cross between F_1_ males and normal females. Surprisingly, microarray clustering showed F_1_ male profile as closest to F_1_ female and F_0_ male profile closest to F_2_ male. Differentially expressed genes in F_1_ males, F_1_ females and F_2_ males showed significant overlap with those caused by PTZ. Interestingly, microarray evidence also led to the identification of upregulated rRNA in F_2_ males. Next, we generated microarray expression profiles of adult testis from F_0_ and F_1_ males. Further surprising, clustering of CNS and testis profiles and matching of differentially expressed genes in them provided evidence of a spermatogenic mechanism in the transgenerational effect observed. To our knowledge, we report for the first time detection of transgenerational spermatogenic inheritance of adult acquired somatic gene expression characteristic. The *Drosophila* systems model offers an excellent opportunity to understand the epigenetic mechanisms underlying the phenomenon. The finding that adult acquired transcriptomic alteration in soma is spermatogenically inherited across generations has potential implications in human health and evolution.

## Introduction

Environmental exposures influence health and disease. Understanding environment-genome interactions is crucial for dissecting the underlying mechanisms. Whereas only a few of the environmental factors that cause disease susceptibility have been shown to promote mutation in DNA sequence, emerging evidence suggests that environmental influences may mainly be mediated by epigenetics, i.e., the processes that lead to changes in gene expression without a change in the DNA sequence [1], [2], [3]. Because epigenetic changes can alter whole-genome expression profiles of various cell types that constitute different tissues and organs, these modifications provide plausible basis for transcriptomic alterations that are associated with various diseases [4], [5]. Epigenetic alterations can be mitotically inherited in somatic cells and can exert long-term effect on gene expression. This mechanism is supposed to underlie risk of developing diseases secondary to prenatal and early postnatal environmental exposures [6], [7], [8], [9]. Importantly, increasing evidence suggests that meiotically heritable epigenetic modifications may also be transgenerationally inherited [2], [10], [11], [12], [13]. Available instances of inheritance of epigenetic transgenerational phenotype are limited to environmental exposures during embryonic and adult gonadal development [14], [15]. Adult exposures can also affect gametogenesis and thereby potentially result in reprogramming of the germline [14]. Although examples of epigenetic effects on gametogenesis exist, it is notable that transgenerational inheritance of environment-induced adult phenotype has not yet been reported [14], [16], [17], [18], [19], [20].

Model organisms have proven highly valuable in understanding epigenetic mechanisms of gene regulation [21]. Vertebrate and invertebrate species share numerous aspects of germ cell behavior, migration and gonadal development [22], [23]. Certain molecular aspects of germ cells and gonadal development are also suggested to be similar in different organisms [22], [23]. Notably, available data suggests that major features of chromatin condensation in *Drosophila* spermatogenesis correspond to those of the epigenetic event in mammalian species [24]. All stages of spermatogenesis, from germline stem cell division to functional sperm production, are present in adult *Drosophila* testes [25]. We selected *Drosophila* to search for evidence of transgenerational epigenetic inheritance of environmental effect following exposure of adult males. Epigenetic codes are considered to be critical in neural plasticity [26]. A *Drosophila* systems model of pentylenetetrazole (PTZ) induced long-term brain plasticity has recently been described [27]. In this model, chronic PTZ treatment of adult males causes alterations in CNS transcriptome [27]. Readily available microarray data showing transcriptomic alteration in heads of PTZ treated male flies motivated us to search for transgenerational effect of the drug, if any, at gene expression level. Transgenerational transcriptomes have earlier been analyzed after embryonic germline exposure [3], [28], [29]. Our focus here was to specifically analyze transgenerational transcriptome after adult spermatogenic exposure. Use of genetically identical animals is required for demonstrating a transgenerational epigenetic effect [30]. We thus used a freshly generated isogenic line of *Drosophila* in our analysis. The wild-type strain was the same that was used previously for developing the PTZ model [27].

## Results

**Figure 1:**
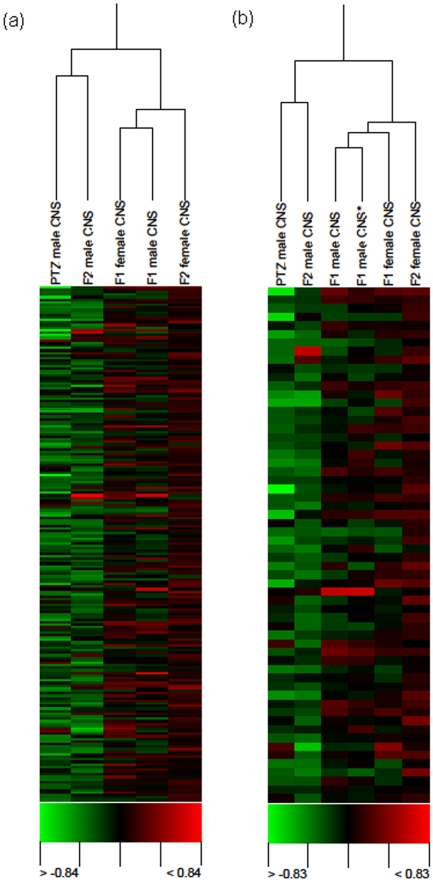
Hierarchical clustering of CNS expression profiles. City Block similarity metric and average linkage methods were used for hierarchical clustering of arrays. The cluster was generated using Acuity 4.0 (Molecular Devices). Each time point represents mean of normalized log_2_ ratio (635/534) of four biological replicates with balanced dye-swaps. Note clustering of PTZ treated males' profile with their grandsons (a). Cluster shown in (b) represents all the profiles in (a) and a freshly generated F_1_ male profile. Reproducibility of expression profiling is evident from similarity between the two F_1_ male profiles (b). PTZ profile shown here was derived from previously reported [27] microarray data related to seven days of drug treatment. * indicates replication set.

The previous study reported microarray gene expression profiles after chronic exposure of unmated males with PTZ for up to seven days [27]. We treated unmated adult F_0_ males with PTZ for seven days and after a washout period of seven days mated them with normally grown females in vials containing normal food (NF). PTZ was never used anytime henceforth. Head gene expression profiles were generated for unmated F_1_ males and virgin F_1_ females resulting from the above cross. Demonstrating transgenerational inheritance secondary to adult exposure requires analysis of F_2_ generation [14]. To raise F_2_, we mated F_1_ males with independently obtained normal females having no history of PTZ exposure in earlier generation. Heads of unmated F_2_ males and virgin F_2_ females were used for generating microarray profiles. Clustering of these profiles along with previously reported [27] PTZ male profile showed similarity between F_2_ male and the latter and between F_1_ male and F_1_ female (**Fig. 1a** **)**. F_2_ female profile was distinct from F_2_ male (**Fig. 1a** **)**. The above result showed that PTZ induced transcriptomic alteration is transmitted to F_2_ generation, with the mode of transmission being complex**.** To examine robustness of this microarray profiling based evidence, we generated another set of F_1_ male CNS microarrays. Importantly, the two F_1_ male profiles clustered together (**Fig. 1b**). This demonstrated that our expression profiles were robust enough for deriving valid inferences. Next, we examined differentially expressed genes in our microarrays. Presence of differentially expressed genes in all the samples – F_1_ males and females, and F_2_ males and females - provided further evidence that PTZ exposure in F_0_ causes genomewide expression perturbation across generations (**Table S1** **, supporting material**). Gene ontology (GO) based analysis showed enrichment of various biological processes in differentially expressed genes (**Table S2** **, supporting material**).

**Figure 2:**
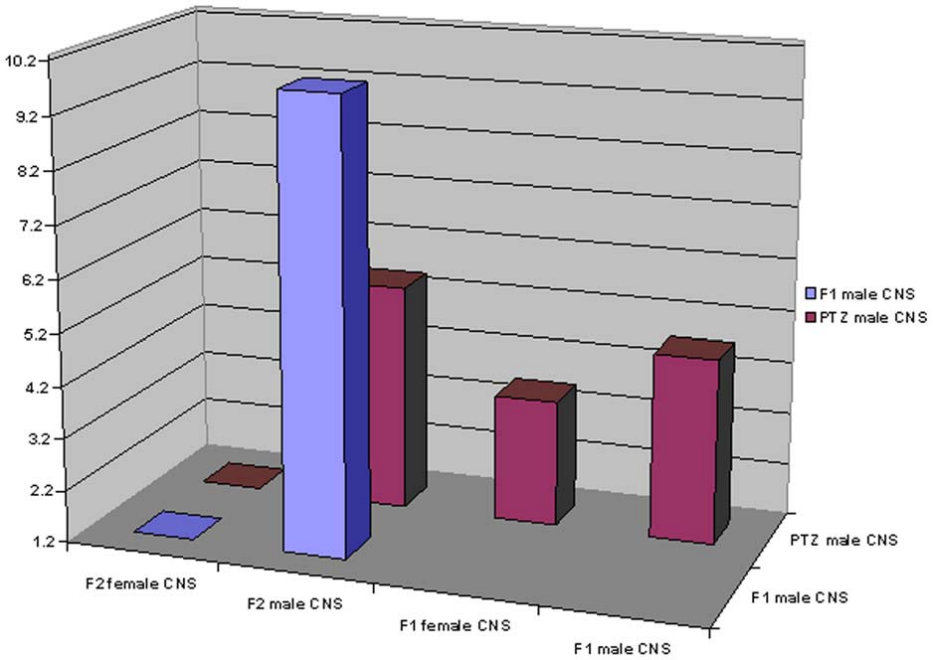
Overlap between differentially expressed CNS genes. PTZ genes used in this analysis was derived from previously reported [27] microarray data related to seven days of drug treatment. PTZ genes were compared to those in F_1_ and F_2_ generations, whereas F_1_ male genes were compared only to F_2_. Hypergeometric distribution *p* values (−log_10_) are plotted on *y*-axis. Note significant overlap (≥1.3) in all except F_2_ female pair-wise comparisons. Differentially expressed genes used in the analysis are listed in Table S1.

We reasoned that if drug-induced transcriptomic perturbation is transgenerationally inherited then statistically significant overlap may be observed between genes regulated by PTZ [27] and those differentially expressed in F_1_ and F_2_. Interestingly, F_1_ males, F_1_ females and F_2_ males showed significant overlap (**Fig. 2**). Besides, genes in F_1_ males significantly overlapped with those in F_2_ males (**Fig. 2**). A lack of overlap in F_2_ females suggested that pattern of inheritance from F_0_ to F_1_ is different than that from F_1_ to F_2_. Cumulatively, our results demonstrated that PTZ's transcriptomic effect is transgenerationally inherited.

**Figure 3:**
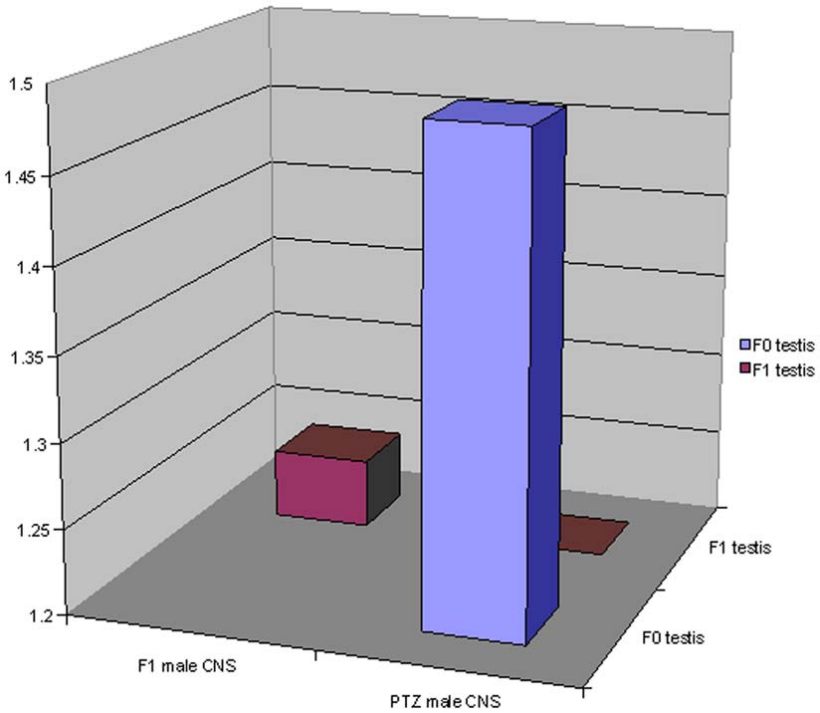
Overlap between differentially expressed testis and CNS genes. PTZ genes used in this analysis was derived from previously reported [27] microarray data related to seven days of drug treatment. PTZ CNS genes were compared to those in PTZ testis (F_0_) and F_1_ CNS, whereas F_1_ testis genes were compared only to F_1_ CNS. Hypergeometric distribution *p* values (−log_10_) are plotted on *y*-axis. Note significant overlap (≥1.3) between PTZ CNS and F_0_ testis genes and an overlap between F_1_ CNS and F_1_ testis genes with borderline significance (1.24). Differentially expressed genes used in the analysis are listed in Table S1 and S3.

**Figure 4:**
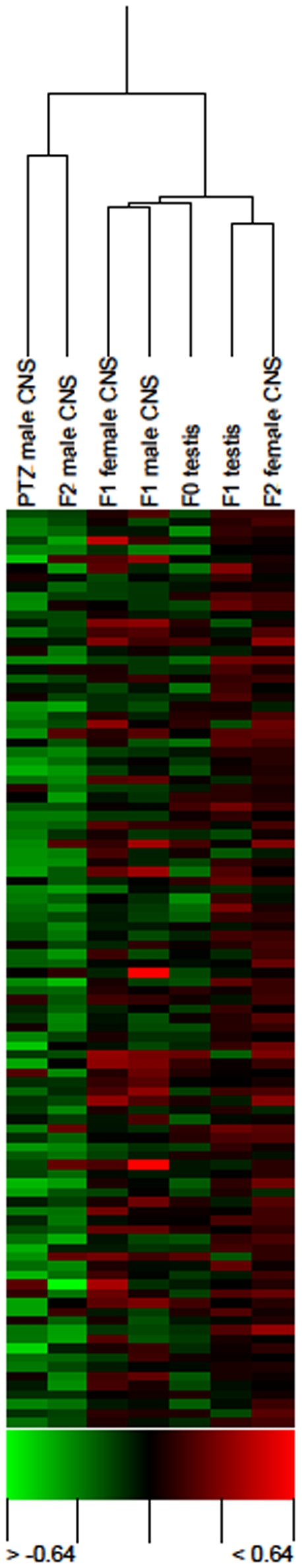
Hierarchical clustering of expression profiles of testis and CNS. City Block similarity metric and average linkage methods were used for hierarchical clustering of arrays. The cluster was generated using Acuity 4.0 (Molecular Devices). Each time point represents mean of normalized log_2_ ratio (635/534) of four biological replicates with balanced dye-swaps. PTZ profile shown here was derived from previously reported [27] microarray data related to seven days of drug treatment.

We next examined if a gametogenic mechanism is involved in the transgenerational effects observed. Majority of transcription in adult *Drosophila* testis occurs in spermatocytes products of which are required for spermatid differentiation [31]. We thus generated microarray expression profiles of F_0_ and F_1_ adult testis. Interestingly, both F_0_ and F_1_ showed transcriptomic alteration. Differentially expressed genes were identified in F_0_ as well as F_1_ testis (**Table S3** **, supporting material**). These genes were found to enrich a few GO biological processes (**Table S4** **, supporting material**). Importantly, PTZ regulated CNS genes [27] showed significant overlap with differentially expressed F_0_ testis genes (**Fig. 3**). Also, genes in F_1_ male CNS and F_1_ testis overlapped with a borderline significance (**Fig. 3**). A lack of overlap between F_0_ and F_1_ testis genes supported the above observation that genomic mechanism underlying F_0_ to F_1_ transmission is different from that of F_1_ to F_2_. To further examine if what we observe is a case of transgenerational spermatogenic inheritance, we clustered CNS and testis microarrays together. Interestingly, F_0_ testis clustered with F_1_ male and F_1_ females whereas F_1_ testis clustered with F_2_ female (**Fig. 4**). Cumulatively, the above results provided evidence that transcriptomic perturbation set off by PTZ in CNS perpetuates to future generations by gametic involvement.

**Figure 5:**
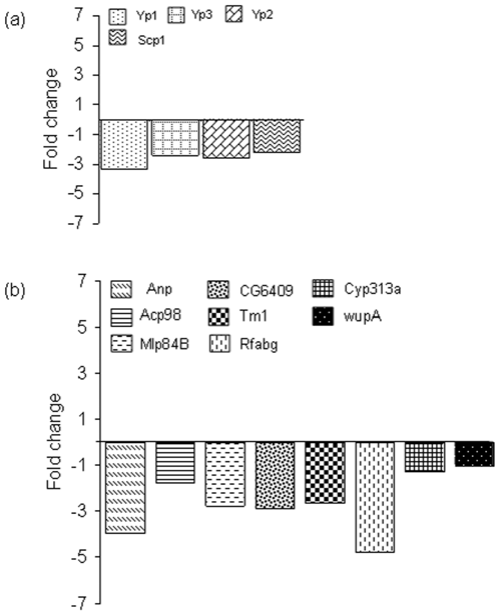
Validation of microarrays using RT-PCR. Genes were either upregulated (a) or not differentially regulated (b) in F_2_ male microarrays. Equal amount of F_2_ male RNA samples from all the four biological replicates used in microarray experiment represented in Figure 1 were pooled together for use in RT-PCR. Fold-change values are plotted on *y*-axis. Note general downregulation of gene expression in RT-PCR.

**Figure 6:**
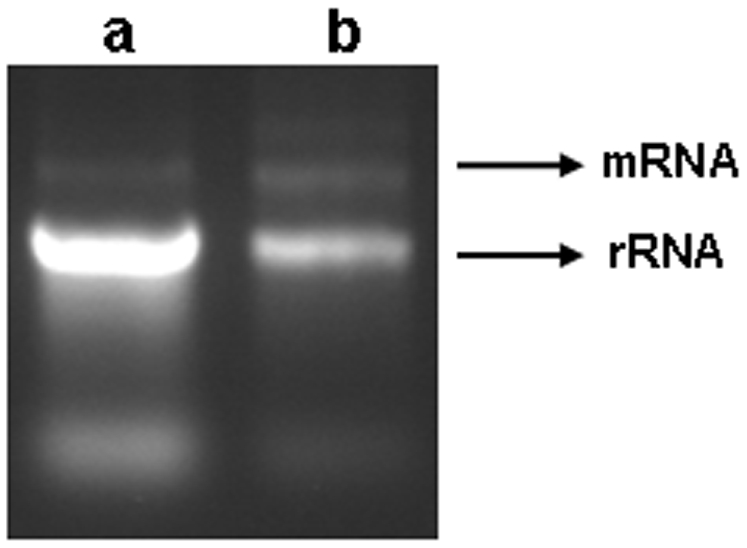
Electrophoretic analysis of F_2_ male CNS total cellular RNA. Equal amount of F_2_ male RNA samples from all the four biological replicates used in microarray experiment represented in Figure 1 were pooled together for electrophoretic analysis. Note higher amount of rRNA in experimental flies (a) compared to control (b).

Microarray expression profiling provided evidence of transgenerational spermatogenic inheritance at overall transcriptomic level. To confirm individual gene expression differences, we selected four genes showing upregulation in F_2_ male microarrays (**Table S1** **, supporting material**) and assessed their expression using quantitative real-time RT-PCR. Intriguingly, all four genes showed downregulation in RT-PCR (**Fig. 5a**). To investigate the discrepancy, we selected eight genes which were not differentially expressed in F_2_ male microarrays (**Table S1** **, supporting material**) and analyzed their expression using RT-PCR. Unexpectedly, these genes in general also showed downregulation (**Fig. 5b**). One possible explanation of this finding could have been that the endogenous control used in RT-PCR, 18S rRNA, may itself be upregulated in F_2_. Most remarkably, comparison of total RNA isolated from heads of control F_2_ and F_2_ adult males with history of PTZ exposure in F_0_ showed higher abundance of rRNA in the latter (**Fig. 6**). This result was surprising. Synthesis of rRNA is known to be upregulated by enriched nutritional conditions [32]. Remarkably, all three genes encoding yolk proteins in *D. melanogaster*, *Yp1*, *Yp2* and *Yp3*, used for nutritional purpose, were among the total 16 upregulated genes in F_2_ male CNS (**Table S1** **, supporting material**). These three genes together represented the most significantly enriched biological process, vitellogenesis, in these males (**Table S2** **, supporting material**). Considering that *Yp1*, *Yp2* and *Yp3* are female specific genes [33], it was interesting to note their expression in males.

## Discussion

We have shown here that exposure of a neuroactive compound to adult *Drosophila* males causes gene expression alteration in CNS of not only the individuals exposed but also their future generations. The genes affected reveal statistically significant overlap across generations. Further, gene expression in adult gonads also shows alterations across generations. The affected genes in testis show some overlap with those in CNS. Transcriptomic similarity thus provides credible evidence that drug induced gene expression alterations leaks to future generations through gametes. Interestingly, we also find that drug exposure causes abnormal expression of rRNA in grandsons of exposed individuals.

Notwithstanding its Lamarckian flavor, transgenerational spermatogenic inheritance has been detected in our search. To our knowledge, this is the first evidence of gametogenic inheritance of adult male acquired somatic characteristic across generations. Given this, our fly model offers an excellent opportunity to further dissect mechanisms underlying the epigenetic inheritance involved. Notably, the model can readily be used as a simple means to test agents for their potential in causing transgenerational effects or erasure thereof. For example, differential expression of rRNA in F_2_ generation may be used as a rapid screen to test agents producing or modifying transgenerational effects.

Although it is difficult to predict the exact mechanism of epigenetic inheritance observed, involvement of some kind of nervous system-gametogenesis connectivity may possibly be hypothesized. It is interesting to speculate here, for example, a role of neuropeptides as a connecting link. These peptides regulate most, if not all, biological processes across animal species including *Drosophila* and humans [34]. Neuropeptides are expressed by neurosecretary cells and may be released into the circulatory system to act as neurohormones [35]. Neuropeptides and their receptors are known to express in germ cells [36], [37]. Neuropeptide mediated synaptic plasticity is known to involve regulation of gene expression and chromatin structure [38]. It is tempting to speculate that inheritance of gene expression alteration induced by brain plasticity interfering drugs is mediated by epigenetic changes in the gametes caused through some kind of nervous system- spermatogenesis axis. Alternatively, a direct effect of environmental agents on gametogenesis, besides CNS, may also be possible.

The evidence that gene expression phenotype acquired by an adult can be transmitted to future generations has obvious implications in human health and evolution. Recent epidemiological evidence supports existence of sex-specific, male line transgenerational responses in humans [39]. The experimental evidence presented here warrants systematic investigations to examine if epigenetic inheritance of environmentally induced characteristics exists in man. A topical example to underscore the necessity of such efforts is the ongoing debate whether the use of cognitive enhancement drugs by normal healthy individuals is ethical [40]. Evidence supporting transgenerational inheritance of effects produced by these drugs would compel the present argumentation in a radically new direction.

## Materials and Methods

### Fly handling and drug treatment

Unless mentioned otherwise, previously described materials and methods were used [27]. In brief, standard fly medium consisting of agar-agar, maize powder, brown sugar, dried yeast and nipagin was used. An isogenic line of *D. melanogaster* Oregon-R strain was used. Standard methods of fly handling and manipulation were followed. Final concentration of PTZ (Sigma-Aldrich) in the fly medium was 8 mg/ml. Unmated males were first treated with PTZ for seven days and then crossed with virgin females in groups seven days after withdrawing PTZ, to obtain F_1_. Unmated males and virgin females of F_1_ generation were collected separately. The F_1_ males were crossed to normally grown females, without any history of PTZ exposure in earlier generation, in groups to obtain F_2_ progeny. Unmated males and virgin females of F_2_ generation were collected separately. For use as control in expression analysis, flies were treated with vehicle (water) instead of PTZ in parallel.

### Microarrays

Unless mentioned otherwise, previously described materials and methods were used [27]. In brief, total RNA was isolated from frozen fly heads or testis using TRI REAGENT (Sigma), according to the manufacturer's protocol. Microarray -cDNA Synthesis Kit, -Target Purification Kit, and -RNA Target Synthesis Kit (Roche) were used to generate labeled antisense RNA. Four biological replicates, with balanced dye-swap, were used for generating expression profiles. Each replicate compared experimental versus control flies. The hybridization mixture was denatured at 65°C and applied onto cDNA microarray slides (14Kv1, CDMC, Canada). Analyzable spots in at least three of four biological replicates performed were retrieved for downstream analysis using SAM 3.0 (Excel Add-In) [41]. Wherever absent in the fourth replicate, the values were imputed using SAM. For cluster analysis, all four values in the entire set were used. City Block similarity metric and average linkage methods were used for hierarchical clustering of arrays. The cluster was generated using Acuity 4.0 (Molecular Devices). For identifying differentially expressed genes, both false discovery rate (FDR) in SAM and fold change (FC) in Acuity were used separately. Previously, microarray profiling using the same methods used here identified reported no gene as differentially expressed below 96% FDR [27]. In the present SAM analysis, genes were considered differentially expressed if found within 20% FDR in SAM. For FC analysis, equal or more than 1.3 FC was used for all profiles except F_1_ testis where 1.2 was used instead of 1.3. Significant match was found between SAM and FC sets of differentially expressed genes. As number of differentially expressed genes identified by either method was smaller, both sets were combined together for further analysis. As differentially expressed genes matched significantly between two replicates of F_1_ male profiles, the two sets were combined together for further analysis. GOTool Box [42] was used to retrieve overrepresented biological processes in up- or down- regulated genes, under the settings, ontology, biological process; mode, all terms; reference, genome; evidence, all-all evidence; species, *D. melanogaster*; GO-stats; statistic test, hypergeometric, Bonferroni adjustment. Hypergeometric distribution probabilities for matching of differentially expressed genes between profiles were calculated assuming population sizes of 12000, approximately the number of unique genes in the microarrays. The full microarray data set has been deposited in the Gene Expression Omnibus (http://www.ncbi.nlm.nih.gov/geo/) under accession series GSE15136.

### Real-Time PCR

Equal amount of RNA samples from all the four biological replicates used in microarray experiment were pooled together for use in RT-PCR. Unless mentioned otherwise, previously described materials and methods were used [27]. In brief, RT-PCR amplification reactions were carried out in an ABI Prism 7700 sequence detection system (Applied Biosystems). ABI gene expression assay IDs used were Dm02151842_g1, Dm01813277_g1, Dm01813506_g1, Dm01813276_g1, Dm02152877_s1, Dm03420546_m1, Dm02361408_s1, Dm02367441_s1, Dm02149362_m1, Dm01825573_m1, Dm02140334_g1 and Dm01828736_m1, for *Anp, Yp1*, *Yp3*, *Yp2*, *Acp98*, *Scp1*, *Mlp84B*, *CG6409*, *Tm1*, *Rfabg*, *Cyp313a1* and *wupA*, in that order.

### Gel electrophoresis

Equal amount of RNA samples from all the four biological replicates used in microarray experiment were pooled together for electrophoretic analysis. Standard methods of RNA agarose gel electrophoresis and ethidium bromide staining were followed.

## Supporting Information

Table S1 Differentially expressed genes in CNS. Up- and down-regulated genes in F1 and F2 male and female CNS are listed. Except F1 male, each set of genes were identified using four biological microarray replicates with balanced dye-swaps. In F1 males, an additional batch of four microarrays were generated. Differentially expressed genes identified using each batch showed significant overlap and were therefore pooled together. See text for further details.
(0.30 MB XLS)

Table S2 Enriched biological processes in differentially expressed genes in CNS. Gene lists provided in Table S1 were used in enrichment analysis. GOTool Box was used to retrieve overrepresented biological processes in up- or down- regulated genes. See text for further details.
(0.13 MB XLS)

Table S3 Differentially expressed genes in testis. Up- and down-regulated genes in individulas exposed to PTZ (F0) and F1 male are listed. Each set of genes was identified using four biological microarray replicates with balanced dye-swaps. See text for further details.
(0.03 MB XLS)

Table S4 Enriched biological processes in differentially expressed genes in testis. Gene lists provided in Table S3 were used in enrichment analysis. GOTool Box was used to retrieve overrepresented biological processes in up- or down- regulated genes. See text for further details.
(0.03 MB XLS)
